# Perforated jejunal diverticula: a case report

**DOI:** 10.1186/1752-1947-4-172

**Published:** 2010-06-07

**Authors:** Joseph S Butler, Christopher G Collins, Gerard P McEntee

**Affiliations:** 1 Department of Surgery, Mater Misericordiae University Hospital, Dublin, Ireland

## Abstract

**Introduction:**

Jejunal diverticula are rare and are usually asymptomatic. However, they may cause chronic non-specific symptoms or rarely lead to an acute presentation.

**Case presentation:**

We report the case of an 82-year-old Caucasian woman presenting with a one-day history of generalized abdominal pain, with three episodes of vomiting. An abdominal X-ray displayed multiple dilated loops of the small bowel. A subsequent computed tomography scan of the abdomen and pelvis revealed a thickening of the duodenum and dilatation of the proximal jejunum. Multiple small bowel diverticula were identified with surrounding pockets of free air adjacent to the jejunal diverticula suggestive of a small bowel perforation. Our patient underwent a laparotomy, which identified multiple jejunal diverticula with two pinhole jejunal perforations and associated fecal contamination. The perforations were repaired with primary closure and extensive washout was performed.

**Conclusion:**

Jejunal diverticulosis in the elderly can lead to significant morbidity and mortality and so should be suspected in those presenting with crampy abdominal pain and altered bowel habits.

## Introduction

Jejunal diverticula are rare with an incidence of less than 0.5% [1]. Pathologically, they are pseudodiverticula of the pulsion type, resulting from increased intra-luminal pressure and weakening of the bowel wall. These outpouchings only contain mucosa and submucosa.

Despite most cases of jejunal diverticulosis remaining completely asymptomatic, complications are reported in 10 to 30% of patients [2-4]. These include chronic abdominal pain, malabsorption, hemorrhage, diverticulitis, obstruction, abscess formation and rarely diverticular perforation.

We present a rare cause of acute abdominal pain with a case of perforated jejunal diverticula. We also review the literature associated with the management of small bowel diverticular disease.

## Case presentation

An 82-year-old Caucasian woman of Irish background, presented to the emergency department with a one-day history of generalized abdominal pain, with three episodes of vomiting. The patient had a past medical history significant for hypothyroidism and hypoalbuminemia secondary to malnutrition.

On physical examination our patient's vital signs were a temperature 36°C, heart rate 105, blood pressure 90/50 and respiratory rate 16 breaths/min. Abdominal examination revealed a generalized abdominal tenderness and signs of peritonitis. Laboratory investigations revealed an elevated white cell count (WCC 18.29 × 10^9^/L), an impaired renal profile (urea 13.2 mmol/L; creatinine 139 μmol/L) and an elevated serum lactate (4.6 mmol/L).

**Figure 1:**
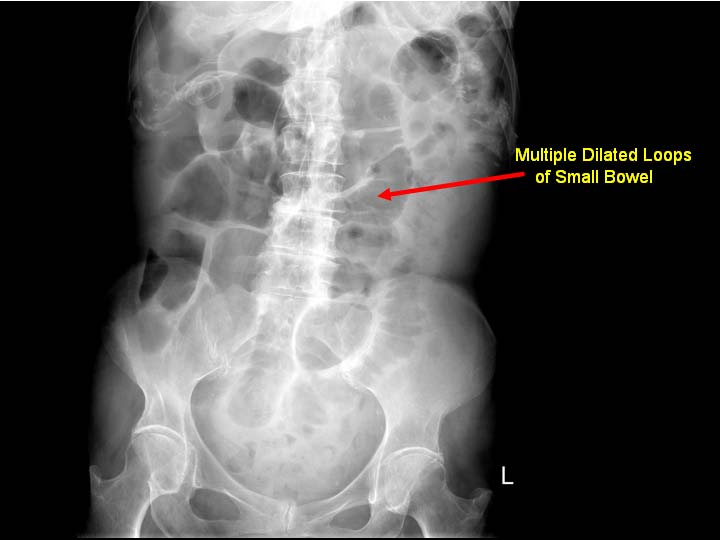
**Abdominal X-ray displayed multiple dilated loops of small bowel**.

**Figure 2:**
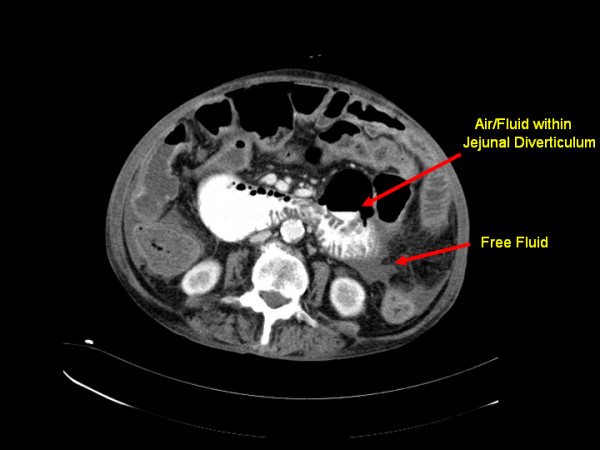
**CT scan of abdomen showing thickening of the duodenum and dilatation of the proximal jejunum**. Multiple small bowel diverticula were identified with surrounding pockets of free air and fluid adjacent to the jejunal diverticula suggestive of a small bowel perforation.

Abdominal X-ray (Figure 1a) displayed multiple dilated loops of small bowel. A subsequent computed tomography (CT) scan of the abdomen and pelvis (Figures 1 and 2) revealed a thickening of the duodenum and dilatation of the proximal jejunum. Multiple small bowel diverticula were identified with surrounding pockets of free air adjacent to the jejunal diverticula suggestive of a small bowel perforation.

**Figure 3:**
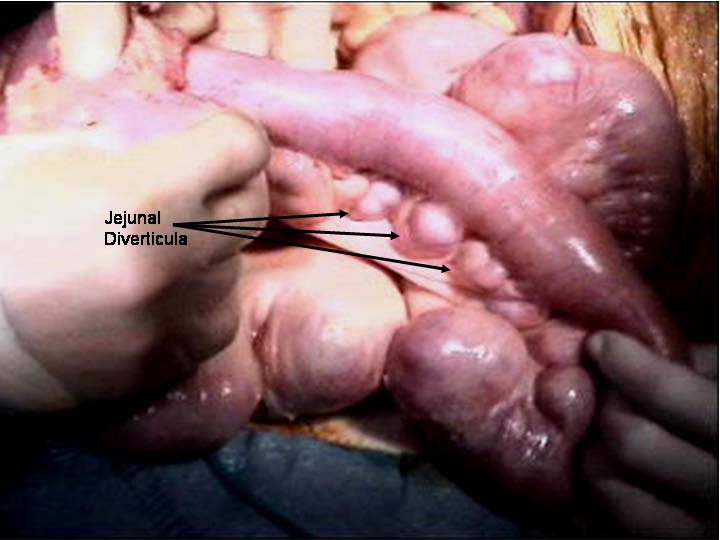
**Intra-operative video images displaying dilated loops of jejunum with multiple jejunal diverticula**.

**Figure 4:**
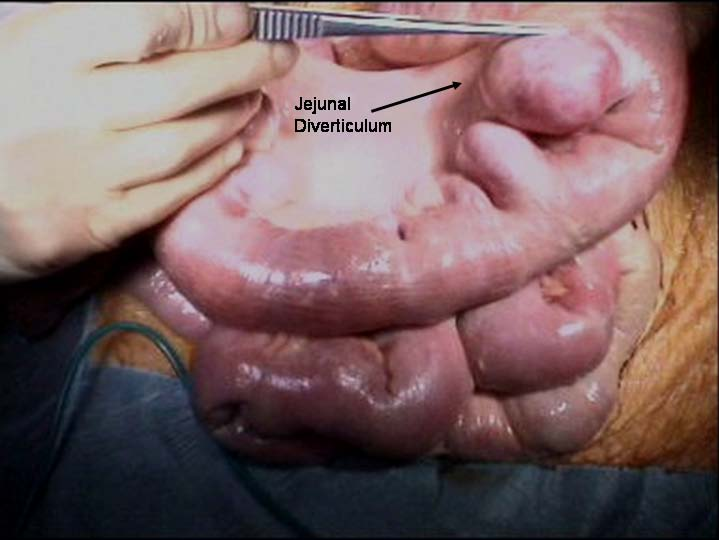
**Intra-operative images of dilated loops of jejunum with multiple jejunal diverticula**.

The patient underwent a laparotomy which identified multiple jejunal diverticula (Figures 3 and 4) with two pinhole jejunal perforations and associated fecal contamination. The two sites of perforation were closed primarily and oversewn. Extensive abdominal washout was performed. Our patient's post-operative course was complicated by an episode of aspiration pneumonia from which she made a full recovery.

## Discussion

Jejunal diverticula are the least common type of small bowel diverticula, with an incidence of less than 0.5% [1]. They are multiple outpouchings of mucosa and submucosa. Although the true etiology of jejunal diverticulosis is unknown, this condition is believed to develop from a combination of abnormal peristalsis, intestinal dyskinesis, and high segmental intra-luminal pressures. These diverticula arise on the mesenteric border where the mesenteric vessels penetrate the jejunum.

Usually, this disorder is clinically silent until it presents with the complications associated with diverticular disease. When symptomatic, patients may describe a vague, chronic abdominal pain of varying severity, localized either to the epigastrium or peri-umbilical region. The only definitive way to confirm jejunal diverticulosis as the primary source of abdominal pain is cessation of symptoms after surgical resection of the involved segment of small bowel. Complications of jejunal diverticulosis warranting surgical intervention occur in eight to 30% of patients [5]. Common acute complications include diverticulitis, bleeding, intestinal obstruction and perforation [6].

Jejunal diverticulosis is a challenging disorder from a diagnostic perspective, with no truly reliable diagnostic tests. Abdominal radiographs and/or chest radiographs may demonstrate evidence of perforation, such as free air under the diaphragm or free peritoneal air; evidence of intestinal obstruction, or evidence of ileus, including multiple air-fluid levels and bowel dilatation. CT may identify thickening or inflammation of the jejunum or localized abscess formation [7,8]. Endoscopic procedures, such as double-balloon enteroscopy and capsule endoscopy, are useful in diagnosing small-bowel disorders [9]. However, these procedures cannot be used in the emergency setting, such as intestinal obstruction or perforation.

Diagnostic laparoscopy can be very useful in investigating patients with a complicated symptomatology. It enables an accurate conclusive diagnosis to be made, avoiding the need for unnecessary laparotomy. In the presence of laparoscopic findings such as perforation, abscesses, and mechanical obstruction, exploratory laparotomy is required with resection of the diseased bowel and primary anastomosis is appropriate.

If the perforation of a jejunal diverticulum causes only localized peritonitis and the patient remains stable, it is has been reported that a trial of non-surgical management with intravenous antibiotics and other supportive measures alongside percutaneous CT-guided aspiration of localized intraperitoneal collections may be suitable and avoid the need for surgery [10]. However, the current treatment of choice for perforated jejunal diverticula causing generalized peritonitis is prompt laparotomy with segmental intestinal resection and primary anastomosis. The extent of the bowel resection depends upon the length of the bowel that is affected by the diverticula and the patient's peri-operative condition [11]. If diverticula are extensive, resection may have to be limited to include only the segment containing the perforated diverticulum and to leave a segment of small bowel that still contains non-perforated diverticula in order to avoid short bowel syndrome [12].

In our case the decision to perform a primary closure was based on the age of our patient and the extent of the diverticulosis, which precluded a safe resection and anastomosis. Jejunal diverticulosis, unlike colonic diverticulosis, tends not to be associated with surrounding diverticulitis and in our case the adjacent tissue was normal in appearance when examined intra-operatively.

## Conclusions

Jejunal diverticula are rare and usually asymptomatic. However, they may lead to chronic non-specific abdominal symptoms or rarely, as displayed by this case, can present as an acute presentation. Jejunal diverticulosis in the elderly can lead to significant morbidity and mortality and so should be suspected in those presenting with crampy abdominal pain and altered bowel habits. Once jejunal diverticulosis has been diagnosed, conservative medical management should be instituted to alleviate symptoms and reduce the risk of complications associated with diverticular disease. Rarely, jejunal diverticular disease may present as intestinal perforation, for which surgical repair is the treatment of choice.

## Consent

Written informed consent was obtained from the patient for publication of this case report and any accompanying images. A copy of the written consent is available for review by the Editor-in-Chief of this journal.

## Competing interests

The authors declare that they have no competing interests.

## Authors' contributions

JSB conceived the study, acquired patient data and drafted the manuscript. CGC critically reviewed the manuscript. All authors (JSB, CGC, GPMcE) contributed intellectual content and have read and approved the final manuscript.
